# Regulation of visceral and epicardial adipose tissue for preventing cardiovascular injuries associated to obesity and diabetes

**DOI:** 10.1186/s12933-017-0528-4

**Published:** 2017-04-04

**Authors:** N. González, Z. Moreno-Villegas, A. González-Bris, J. Egido, Ó. Lorenzo

**Affiliations:** 1 Renal, Vascular and Diabetes Laboratory, Instituto de Investigaciones Sanitarias-Fundación Jiménez Díaz (IIS-FJD), Universidad Autónoma, Madrid, Spain; 2 Spanish Biomedical Research Centre in Diabetes and Associated Metabolic Disorders (CIBERDEM) Network, Madrid, Spain

**Keywords:** Visceral adipose tissue, Epicardial adipose tissue, WAT, BAT, PPARγ, Statin, Incretin

## Abstract

Nowadays, obesity is seriously increasing in most of the populations all over the world, and is associated with the development and progression of high-mortality diseases such as type-2 diabetes *mellitus* (T2DM) and its subsequent cardiovascular pathologies. Recent data suggest that both body fat distribution and adipocyte phenotype, can be more determinant for fatal outcomes in obese patients than increased general adiposity. In particular, visceral adiposity is significantly linked to long term alterations on different cardiac structures, and in developed forms of myocardial diseases such as hypertensive and ischaemic heart diseases, and diabetic cardiomyopathy. Interestingly, this depot may be also related to epicardial fat accumulation through secretion of lipids, adipokines, and pro-inflammatory and oxidative factors from adipocytes. Thus, visceral adiposity and its white single-lipid-like adipocytes, are risk factors for different forms of heart disease and heart failure, mainly in higher degree obese subjects. However, under specific stimuli, some of these adipocytes can transdifferentiate to brown multi-mitochondrial-like adipocytes with anti-inflammatory and anti-apoptotic proprieties. Accordingly, in order to improve potential cardiovascular abnormalities in obese and T2DM patients, several therapeutic strategies have been addressed to modulate the visceral and epicardial fat volume and phenotypes. In addition to lifestyle modifications, specific genetic manipulations in adipose tissue and administration of PPARγ agonists or statins, have improved fat volume and phenotype, and cardiovascular failures. Furthermore, incretin stimulation reduced visceral and epicardial fat thickness whereas increased formation of brown adipocytes, alleviating insulin resistance and associated cardiovascular pathologies.

## Background

In 2015, the World Health Organization estimated a worldwide population of 2.3 billion of overweight individuals and more than 700 million of obese adults (http://www.who.int/topics/obesity/en/). In high-income countries, the overall rates are more than four times than those detected in lower and lower-middle income countries, though obesity is dangerously growing in Southeast Asia and Latin America. In addition, this dread is not restricted to adulthood, since at least 41 million children are obese or overweight [1]. Obesity has emerged as one of the most critical global health care problems, being largely associated with multiple pathologies such as insulin resistance, type-2 diabetes mellitus (T2DM), metabolic dysfunction, and several cardiovascular injuries (i.e., acute myocardial infarct, diabetic cardiomyopathy, atherothrombosis), which may lead to heart failure (HF) and death. In this sense, a weight loss can be potentially reached by lifestyle modifications, and pharmacological and/or surgical interventions, and this may be linked to improvements in cardiovascular function [2]. Interestingly, the most efficient therapies against the development of cardiometabolic pathologies may target the altered composition and distribution of fat stores. In the present review, we will examine the effects and mechanisms of action of excessive visceral fat storing on HF, especially through its influence on epicardial fat depots. In addition, we will also discuss conventional and prospective interventions in obese and T2DM patients to reduce and distribute visceral and epicardial fat repositories in relation with the associated cardiometabolic risk.

## Fat distribution and composition

Obesity, particularly in those patients with higher body mass index (BMI) levels (≥30 kg/m^2^), is linked to increased cardiovascular mortality compared to normal weight (BMI = 18.8–24.9 kg/m^2^) [3]. However, the heterogeneity of fat composition [white (*WAT*), brown (*BAT*) and beige/brite/brown-like (*bAT*) adipose tissues] and the distribution of these depots, can be more crucial for the development of cardiometabolic disruptions [4, 5]. In physiological conditions, the presence of *WAT* and *BAT* in various stores of adipose tissue suggests a direct transformation of differentiated pre-adipocytes (Myf5^−^ and Myf5^+^, for *WAT* and *BAT*, respectively) into mature cells with different morphological and functional characteristics according with location [6]. In general, *WAT* accumulates excess of energy as single lipid droplets of triglycerides (TAG) within its adipocytes, which express high levels of leptin and exhibit a few variable number of mitochondria (Table 1). *WAT* weight generally represents as much as 20% of body weight on normal adults and primarily acts as a storage site for fat, preserving supplementary calories for use in times of scarcity [5]. On the other hand, *BAT* can store lipids in multiple small vacuole inside its smaller multi mitochondrial brown adipocytes. *BAT* generates non-shivering thermogenesis and energy dissipation by oxidation of glucose and fatty acids, and activation of the mitochondrial transporter uncoupling protein-1 (UCP1), which deviate electron transfer from ATP synthesis to dissipate protons across the inner mitochondrial membrane, producing heat [7].

**Table 1:** Differences and similarities between the various adipose tissue depots

	
Body location	Gluteofemoral, subcutaneous and VAT	Inguinal and neck	Suprarenal, paravertebral and supraclavicular
Morphology	Big size and with a single LD	Intermediate size and with multiple LD	Variable size, vascularized and with multiple small LD
Mitochondria	+	++	+++
Function	Energy storage	Adaptive thermogenesis (UCP-1)	Non-shivering thermogenesis and energy dissipation
Obesogenity and diabetogenity	Positive	Negative	Negative

Body fat is stored as *WAT*, *bAT* or *BAT*. These depots can be distinguished by their location, size, mitochondria content, and function, playing diverse roles in obesity and T2D. Importantly, *WAT* could be browned to *bAT* and *BAT* by several approaches. *LD* stands for lipid droplet(s)

*WAT* is found in gluteofemoral (found in the lower-body parts), subcutaneous (immediately below the dermis), and visceral locations [8] (Table 1). This visceral adipose tissue (VAT) surrounds the inner organs and can be divided in intraperitoneal [omental (for stomach and spleen), mesenteric (for intestine) and epiploic (for colon)], retroperitoneal (surrounding the kidneys), gonadal (adhered to the uterus/ovaries or epididymis/testis), and pericardial or epicardial adipose tissue (around heart). Interestingly, this epicardial adipose tissue (EAT) is correlated to VAT and can play an essential role in cardiac function and homeostasis (see later). By contrast, *BAT* has relatively large depots in infancy but small volume dispersed throughout *WAT* stores in adults, where it generally locates in suprarenal, paravertebral and supraclavicular regions, as well as areas near large vessels [9]. Finally, the third adipose repository, *bAT*, has mixed features of both *WAT* and *BAT*. *bAT* is intermediate in size and number of mitochondria and its beige adipocytes could be originated from multipotent pre-adipocytes located in various *WAT* depots, or from trans-differentiation of a white adipocyte into a beige (and later brown) adipocyte (i.e., *WAT*-to-*BAT* trans-differentiation or “fat browning”) (Table 1). *bAT* expresses also UCP-1 in humans and is mainly sited in inguinal and neck regions to function for adaptive thermogenesis [10].

## Visceral adipose tissue overload and cardiovascular risk

In multiple regression analyses, the traditional cardiovascular risk markers BMI, low-density lipoprotein cholesterol (LDL-C), and family history of T2DM, were long-term predictors of accumulation of VAT and subcutaneous fat volumes, from young towards middle age healthy men [11]. However, excessive VAT was more pathogenic than overloaded subcutaneous fat, since VAT closely linked to cardiometabolic abnormalities [12]. In this sense, VAT accumulation has been correlated with increasing incidence of T2DM, T2DM-associated chronic low-grade inflammation, atherogenic dyslipidemia, and hypertension [13, 14]. VAT was also an independent negative marker of peripheral insulin sensitivity [15], which associated with components of the metabolic syndrome (i.e., hyperglycemia, hypertriglyceridemia, and low HDL-C) [16]. Importantly, several clinical studies have linked VAT deposition with HF. The Multi-Ethnic Study of Atherosclerosis (MESA) reported that VAT independently associated with augmented left ventricular concentricity and hypertrophy [17]. The Health ABC [18] and Cardiovascular Health [19] studies demonstrated a positive relationship between VAT and HF, independently of BMI, waist circumference and the waist-hip ratio, as anthropometric surrogates values for predicting VAT accumulation [20].

**Fig. 1:**
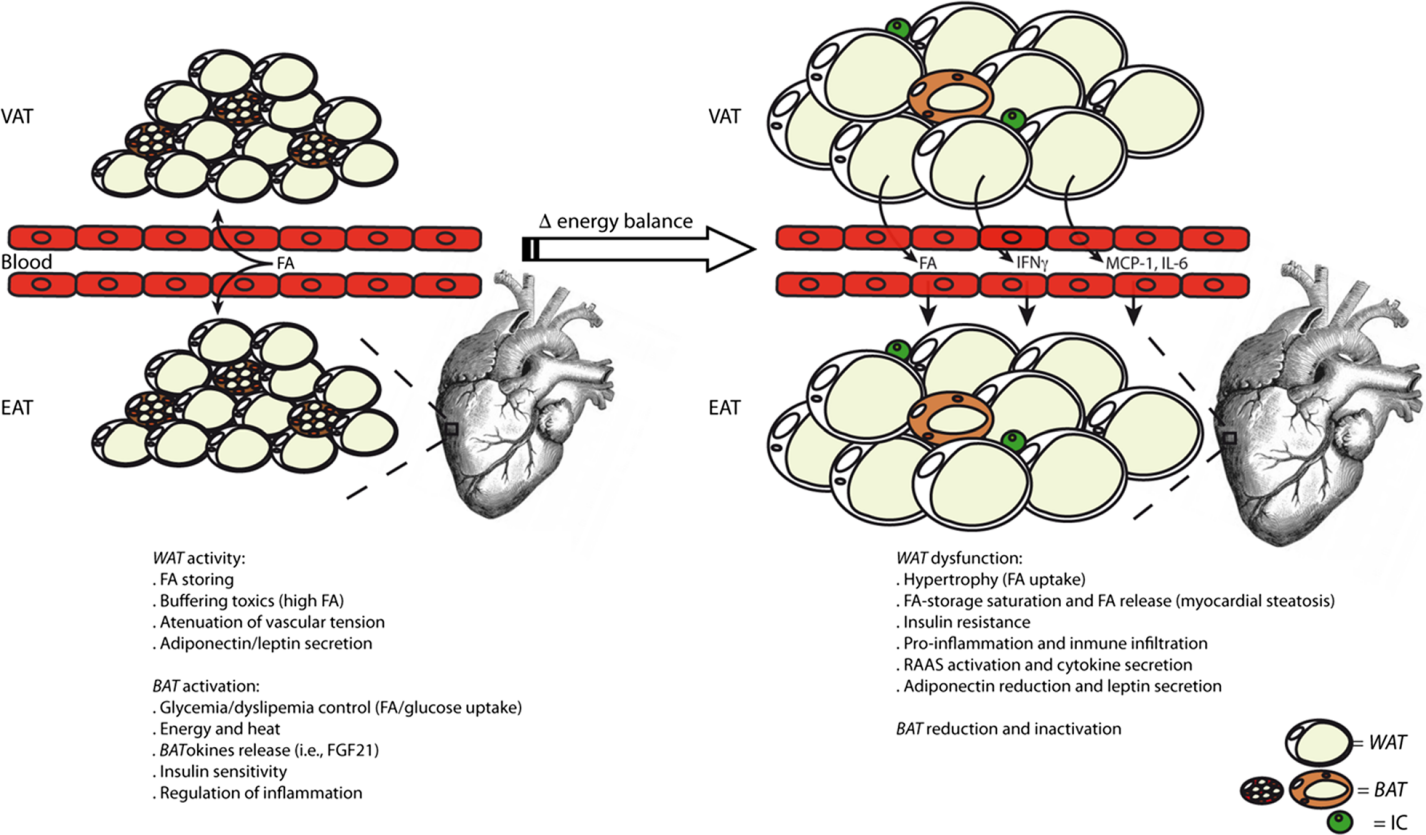
VAT and EAT alterations under obesity and T2DM. In non-obese and non-T2DM subjects, *WAT* and *BAT* deposits in both VAT and EAT serve as storages and buffers for fatty acids (FA), attenuators of glycaemia and dyslipidaemia, and as controllers of vascular tension and inflammation. However, under abnormal or excessive fat accumulation, *WAT* and *BAT* depots become thicker and dysfunctional. *WAT* hypertrophies and it saturates, releasing FA and pro-inflammatory factors (cytokines, chemokines, RAAS) towards circulation and myocardium, leading to immune cells (IC) infiltration, myocardial steatosis and insulin resistance. *BAT* becomes reduced, atrophied and inactive (UCP-1 negative), losing its protective anti-glycemic/dyslipemic and anti-inflammatory effects

After chronic positive energy balance, *WAT* adipocytes in VAT lead to free fatty acid (FFA) uptake and accumulation (Fig. 1). *WAT* expansion in VAT triggered the expression of pro-inflammatory adipokines, oxidative stress and renin-angiotensin-aldosterone system (RAAS) activation. Hypertrophic but not hyperplastic adipocytes, were associated with insulin resistance [21]. Thus, VAT become dysfunctional, dysregulating also adipocyte apoptosis and increasing autophagy [22]. The propensity to preferentially accumulate *WAT* in VAT stores under conditions of excess energy intake is highly variable from one individual to another. The main etiological factors include age, gender, sex and growth hormones, the endocannabinoid and hypothalamus–pituitary–adrenal systems, glucocorticoids, nutritional factors, and physical activity [23]. Nevertheless, accumulation of fat may saturate VAT capacity. The resultant failure of VAT to store TAG could result in ectopic deposition of toxic fatty acids species (i.e., diacylglycerol, ceramide) in extra-adipose tissue such as myocardium, leading to an increase of EAT thickness [24]. Importantly, the amount of VAT correlates with the volume of EAT, and thus, significant weight loss in obese patients has been associated with noteworthy reduction in the EAT volume [25].

## Epicardial adipose tissue and cardiac function

EAT is physically next to the myocardium within the lateral wall of the right ventricle and the anterior wall of the left ventricle, surrounding the right coronary artery and the left anterior descending coronary artery [26]. Thus, both EAT and myocardium share the same microcirculation. Computed tomography allows quantification of EAT, which correlates with advancing age and is usually larger in men than in women [25]. EAT displays high rates of *WAT* lipogenesis but also shows high degrees of *WAT* lipolysis, serving as local TAG store in metabolic stress and as a buffer for high toxic levels of FFA, in both myocardium and arteries [24] (Fig. 1). EAT may also assist a supportive attenuator of vascular tension, participating in vessel remodelling and paracrine responses by releasing specific molecules for cardiovascular protection. In this sense, adiponectin and adipocyte-derived relaxing factors are discharged to decline contractile and vasoconstrictive effects through endothelium-dependent (i.e., increasing NO/ET-1 ratio) or independent (i.e., reducing cell hyperpolarization and ROS production) mechanisms [27, 28]. Moreover, resident macrophages in EAT can release anti-inflammatory cytokines such as IL-10 [29]. Importantly, EAT transcriptome unveiled that this depot is enriched with genes involved in coagulation, endothelial function, phospholipase activity, apoptosis, and immune signaling [30].

Thus, EAT can protect against myocardial stress, hypertension and local inflammation, and may even function as a *BAT* store by protecting adjacent tissues from hypothermia because of its small thermogenic adipocytes [31]. In this regard, specific *BAT* genes such as PR domain containing-16 (PRDM-16), PPAR-γ coactivator 1-α (PGC1α), and UCP-1, are more expressed in EAT than in other fat locations [32]. However, in obesity and T2DM, EAT becomes thicker and dysfunctional, promoting cardiovascular injuries, such as coronary artery disease (CAD) [30, 33].

## EAT dysfunction and cardiovascular risk

As VAT, EAT is also subjected to the maladaptive adipocyte biology of obesity, which is characterized by hypertrophy, failure to store TAG, increased lipolysis, and inflammation. A systematic review of several meta-analysis studies showed that EAT was correlated with plasma TAG, fasting glucose, and metabolic syndrome, and it was linked to high systolic blood pressure and CAD [33].

Adipocyte tissue in EAT undergoes FFA uptake, macrophage infiltration, and endothelial cell activation at the heart [34]. A surplus of FFA uptake leads to FFA accumulation through expansion of adipocytes. EAT reached 7.5 mm in thickness in the human metabolic syndrome compared to 4.0 mm in control patients, and this accumulation disturbed insulin resistance in a similar fashion as central fat [35]. Metabolically healthy obese individuals showed more but smaller-sized adipocytes than obese patients with metabolic disorders [36]. Also, rheumatic patients treated with steroids, which are known to imitate some effects of metabolic syndrome, develop thickening of EAT [37]. Thus, threshold values for high-risk echocardiographic EAT measures (over 9.5 and 7.5 mm in men and women, respectively) may be of help for cardiometabolic risk stratification in obese and T2DM subjects [38]. However, EAT quantification is challenging in real clinical practice because of lack of sensitivity and specificity. Imaging acquisition during breath holding and interference of heart beats, water content and fat droplets from parenchymal cells, as well as the biophysical cardiac properties (relaxation times) and field inhomogeneity, can lead to confounding effects and high noise for diagnosis [39]. In this sense, at least in Korean men, increased EAT thickness around the left main coronary artery was not associated with the prevalence of diabetes [40].

Nevertheless, EAT could play an endocrine role over the heart. Dysfunctional adipocytes expressed high levels of pro-inflammatory factors (i.e., IFNγ) that enhanced the pro-inflammatory response of infiltrated immune cells, such as dendritic cells, macrophages, T- and B-cells, and eosinophils [41]. Moreover, accumulated FFA in EAT stimulated macrophages via Toll-like receptor-4 activation, and these macrophages activate pro-inflammatory NF-κB to overexpress chemotactic cytokines (i.e., MCP-1, IL-6) [42]. Consequently, proteomic analysis revealed high levels of anti-oxidant GSTP1, PDIA1, and PGAM1 in EAT compared to subcutaneous adipose tissue in patients with cardiovascular diseases (i.e., cardiomyopathy and CAD), suggesting that EAT suffers greater oxidative stress due to myocardial stress [43]. Then, local inflammation activates resident anti-inflammatory M2 macrophages to pro-inflammatory M1 macrophages, which stimulate cardiac endothelial cells to release more cytokines that reduce insulin signaling in EAT [24]. In this regard, glucose and lipid metabolisms have been shown impaired in EAT of both diabetic and non-diabetic patients with HF [44]. Glucose transport, as well as FFA uptake and re-esterification are decreased, whereas lipolysis is augmented [45]. In addition, anti-inflammatory/-atherogenic adipokines released from EAT (i.e., adiponectin) are also decreased under obesity, contributing to metabolic diseases, and HF [46] (Fig. 1). Particularly, omentin-1, a novel EAT-derived circulating anti-inflammatory and insulin sensitizer adipokine, was reduced in patients with CAD [47].

In addition, EAT can release FFA in the proximity and around the coronaries arteries (perivascular fat), modulating vascular responsiveness to vasoactive agents [48]. EAT may turn into an adverse lipotoxic, pro-thrombotic, and pro-inflammatory organ, being considered a risk factor for CAD and CAD severity [49]. In this regard, EAT-released glycoprotein orosomucoid is an indicator of pro-inflammatory endothelial dysfunction in patients with T2DM or CAD [50]. Also, EAT can discharge FFA into the bloodstream, disturbing vascular homeostasis and endothelial dysfunction, and leading to CAD and hypoxia [51, 52]. In addition, the local RAAS is activated in EAT and contributes to vasoconstriction, inflammation, and following cardiovascular injury [53] (Fig. 1). Finally, due to anatomic proximity of EAT and myocardium and absence of a dividing fascial plane, EAT may also play a key role in myocardial steatosis [24]. The heart possesses an endogenous TAG depot of ≤1.0% organ mass in healthy lean individuals [54]. However, myocardial TAG stores are increased 2 to 4-fold in T2DM and obese patients, which is associated with cardiac hypertrophy and impaired diastolic function [54]. Myocardial steatosis promotes also hypoxia and apoptosis, which strength inflammation [55]. In this line, a bunch of forty-two pro-apoptotic genes (including TNFα and p53) were upregulated in EAT from patients with cardiovascular injuries [56].

## Role of *BAT* on cardiovascular pathophysiology

Remarkably, fat accumulation as *BAT* may be considered an alternative mean to reduce cardiometabolic risk in obesity and T2DM. Despite its small relative size, *BAT* is highly vascularized and constitutes an important glucose, fatty acid, and triacylglycerol-clearing organ, and such function could potentially be used to alleviate dyslipidaemias, hyperglycemia, and insulin resistance [57]. Furthermore, *BAT* influences cardiovascular physiology by releasing factors that regulate vascular tone and both systemic and cardiac metabolisms [9]. *BAT* stimulation by cold, adrenergic signaling and activators such as thyroid hormones, retinoid, leptin, BMPs, and natriuretic peptides, enhances fatty acid availability for mitochondrial degradation [58, 59] (Fig. 1). Interestingly, *BAT* lipolysis of stored TAG not only provide an important source of energy but also activate tissue-specific FFA-receptors [53]. Certain adipocyte-specific branch FFA released from *BAT* diminished adipose tissue inflammation and improved glucose tolerance in obese mice [59, 60]. In this regard, the expression of insulin-sensitive glucose transporter Glut4 has been demonstrated higher in *BAT*, compared to *WAT* [59], and specific cytokines discharged from *BAT* and termed “*BAT*okines”, possess glucose-sensitivity proprieties. For instance, cold-activated *BAT* secreted fibroblast growth factor-21 (FGF-21) to recover metabolic lipid and glucose equilibrium and leading to cardio-protection in experimental cardiac hypertrophy and ischemia. Also, administration of FGF21 in humans improved hyperlipidemia by lowering plasma TAG and LDL-C levels, while increasing HDL-C levels [61]. Neuregulin-4, a *BAT*okine induced during *WAT*-to-*BAT* trans-differentiation, protected against insulin resistance and myocardial ischemia of T2DM mice [62]. Finally, the nerve growth factor (NGF) promoted pro-survival in ischemic cardiomyocytes and diabetic isolated hearts [63].

However, a negative correlation between obesity and levels of *BAT* volume and activity has been recently stated [64] (Fig. 1). In some South Asians populations, the lower amount of *BAT* can explain their frequent metabolic and cardiovascular disorders such as obesity, insulin resistance, T2DM, and dyslipidemia [65]. A reduced activity of *BAT* may predispose subjects to T2DM not only by increasing obesity, but also through a direct pro-diabetic mechanisms, such as by reduction of glucose uptake [59, 64]. *BAT* in obese/T2DM mice was also less vascularized than in wild type, and their brown adipocytes were larger, unilocular, and mostly UCP1-negative [66]. Thus, conservation of *BAT* depots with an anti-obesity phenotype may be suggested for therapeutic interventions against cardiovascular pathologies in obese and T2DM patients.

## Anti-obesity strategies and reduction of cardiovascular risk

A major goal in the therapeutic field of obesity and related cardiovascular disorders is the development of effective treatments to balance the volume of *WAT* and *BAT*, in VAT and EAT stores.

### Non-pharmacological reduction of WAT

**Fig. 2:**
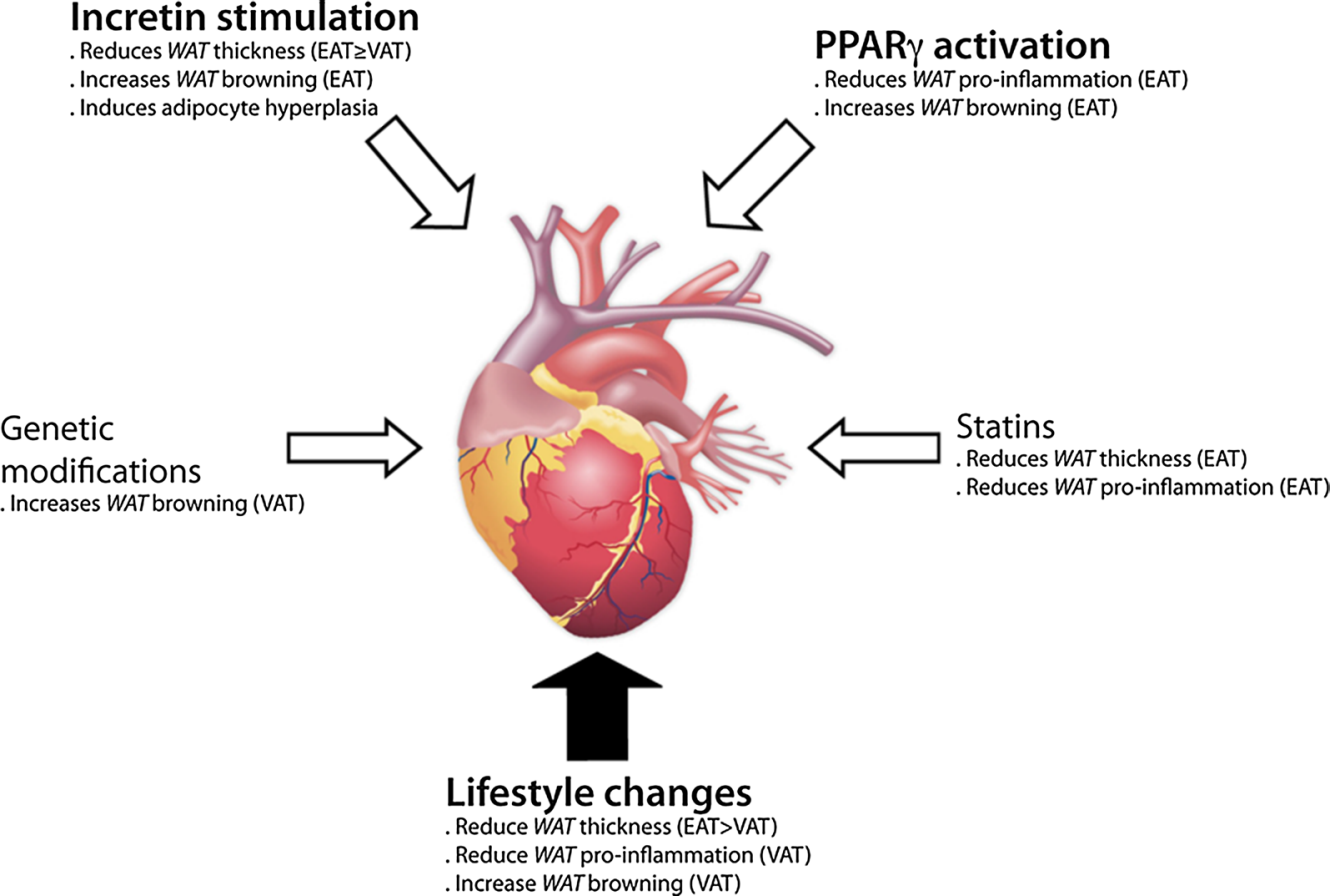
Prospective fat-modulating interventions for cardiovascular dysfunction in obesity and T2DM. In addition to lifestyle modifications on diet and exercise, cardiovascular complications in patients with increased VAT and EAT may be treated with statins or genetic manipulations (focused on USF1, CIDE-A, PGC1a, UCP-1, PRDM-16 or miR-125b-5p) to decrease *WAT* thickness/pro-inflammation in EAT or increase *WAT* browning in VAT, respectively. More significant, PPARγ agonists could promote these effects particularly in EAT, and additionally, incretin stimulation might also induce adipocyte hyperplasia, and subsequent insulin sensitivity

Changes in nutritional or physical activity are the mainstay intervention for overweight, obese and T2DM patients [25]. In moderate and severe obese patients, a weight loss induced by low-calorie diets and exercise showed reductions of BMI, VAT and EAT (Table 2). Interestingly, EAT shrink in a higher proportion than overall adiposity, and this was significantly associated with cardio-protection [67, 68]. Also, aerobic exercise training significantly increased adiponectin secretion independently of the dietary glycemic index and inversely correlated with VAT shortening [69]. In in vitro assays, a low-calorie diet triggered changes in the secretome of human adipocytes by decreasing secretion of *WAT*-released pro-inflammatory adipokines [70] (Fig. 2). Furthermore, in selected obese patients with BMI ≥35, a bariatric surgery intervention may be also recommended. Interestingly, after 6–12 months of laparoscopic Roux-en-Y gastric bypass, obese subjects exhibited a substantial decrease in EAT accompanied with VAT, BMI, waist circumference, and cardiovascular risk factors (i.e., total cholesterol, TAG and fasting blood sugar) [71]. Although EAT loss was lower and more limited than VAT, obese patients exhibited higher secretion of adiponectin and leptin, and lessen *WAT*-related pro-inflammatory adipokines [72, 73]. Intriguingly, the underlying mechanisms of weight loss after bariatric interventions could be more dependent on alteration in gut hormone production [74], neural signalling [75], and glucose/lipid metabolism [76], than those mechanisms related with nutrient absorption.

**Table 2:**
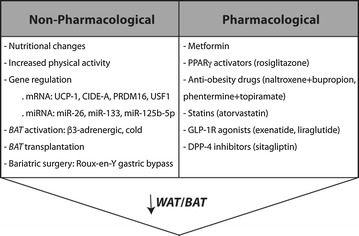
Non-pharmacological and pharmacological strategies to reduce the *WAT*/*BAT* ratio in VAT and EAT depots

By changes in lifestyle and specific miRNA/gene expression, activation or transplantation of *BAT*, and bariatric surgery, the *WAT*/*BAT* ratio can be decreased. Metformin, PPARγ activators, anti-obesity drugs, statins, and more safely, incretins (stabilized by GLP-1R agonists and DPP-4 inhibitors), could also help to reduce this ratio

### Non-pharmacological stimulation of BAT

Supraclavicular *BAT* was associated with less obesity and a more favourable metabolic profile in patients with cardiovascular diseases [77], meanwhile severe *BAT* lipoatrophy aggravated the atherosclerotic process in insulin receptor knockout mice [78]. Consequently, increasing *BAT* formation and activity may account for novel strategies against obesity and its related cardiometabolic pathologies. In this regard, activation of *BAT* by β3-adrenergic receptor increased intracellular lipolysis and subsequent replenishment of lipids through de novo lipogenesis and uptake of TAG and cholesterol from circulation. Thus, hyperlipidemic mice were protected from atherosclerosis [79]. Moreover, *BAT* activation could improve insulin sensitivity via increasing glucose oxidation and lessening body fat mass [80]. Interestingly, high-fat diets stimulated browning capacity of *WAT* in the retroperitoneal depot by stimulating UCP-1 and CIDE-A (cell death-inducing DFFA-like effector-A) expression, likely, as a compensation mechanism [81]. Also, micro-RNAs such as miR-26, miR-27, mir-30, miR-34a, miR-106b, miR-133, miR-155, miR-193-365, miR-196 and miR-378 have been involved in the control of *bAT* and *BAT* formation and function in mice [82].

Therefore, regulation of specific genes or miRs could be used for stimulation of *BAT* browning in obese and T2DM subjects (Fig. 2). Laurila et al. demonstrated that lacking upstream stimulatory factor 1 (USF1) activated *BAT* in obese/T2DM mice, and promoted protection against dyslipidaemia, obesity, insulin resistance, and atherosclerosis. These data were also confirmed in subjects carrying a mutation in USF1 [82]. Also, steroid glycosides as ginsenoside Rb1 improved glucose and lipid metabolisms and reduced body weight in obese animals by up-regulating PRDM-16, PGC1α, and UCP-1 expression and *WAT* browning [83]. Transgenic overexpression of PRDM-16 in subcutaneous *WAT* protected mice from diet-induced obesity and insulin resistance [84]. In addition, injections of an miR-125b-5p inhibitor directly into *WAT* increased β3-adrenoceptor-mediated induction of UCP-1 and *BAT* browning [85].

Furthermore, changes in nutritional or physical activity can also influence on *BAT* in obese and T2DM individuals (Fig. 2). A potential increase of *BAT* volume and activity has been postulated with dietary compounds such as vitamin-A and fish oil [86], or exercise training [87]. However, other researchers have demonstrated no change or even decreased *BAT* activity after exercise [88, 89]. Similarly, the effect of bariatric surgery (i.e., Roux-en-Y gastric bypass or sleeve gastrectomy) in decreasing EAT, is controversial, with a certain variability in the grade of EAT shrinking among the studies [73]. Moreover, activation of *BAT* has been observed in 40% patients following 1 year of laparoscopic adjustable gastric banding surgery [90]. In this line, experimental *BAT* transplantation also headed successful outcomes. This procedure augmented intrinsic expression and activity of thermogenic genes in *BAT* of obese and T2DM mice, and stimulated adiponectin and fatty acid oxidation genes in their *WAT*. *BAT* transplantation additionally improved glucose tolerance and decreased insulin resistance, contributing to reduction of liver steatosis and body weight [91]. However, in humans, the amount of *BAT* is estimated to be less than 0.1% of body weight, which is five times lower than that of mouse [92], and *BAT* seems less prone to be activated, at least by cold exposure, in obese than in lean subjects [93].

Therefore, we know that *WAT* accumulation in VAT and EAT is harmful for obesity and related-cardiovascular diseases, and that reduction of these stores or their browning to *BAT* by changes in nutritional and physical activity can be advantageous. However, hormone production, neural signalling, nutrient absorption and glucose/lipid metabolism could be exclusive for each patient, and unknown (epi)genetic predisposition to obesity and microbiome, may also individually disturb fat storing [94]. Hence, until research progresses on these influencing factors and personalized medicine improves, specific pharmacological approaches could be used to modulate *WAT* and *BAT* activity against obesity.

### Pharmacological reduction of the WAT/BAT ratio

The most noteworthy treatment for T2DM, metformin, decreased VAT volume, activated *BAT* and selectively enhanced clearance of VLDL lipoproteins into *BAT* in obese mice [95] (Table 2). Metformin markedly lowered body weight, plasma cholesterol and TAG, and increased HDL-C levels in obese subjects [96]. However, weight loss could not be achieved in all populations, and fat liver and markers of inflammation and thrombosis were not alleviated [97]. On the other hand, anti-obesity drugs usually work by suppressing appetite, inhibiting fat absorption, or increasing energy consumption and thermogenesis. Unfortunately, some of them (i.e., dexfenfluramine, fenfluramine, sibutramine) have been withdrawn from market because of cardiotoxic side effects [98] Similarly, a PPARγ activator (pioglitazone) was associated with a reduction of pro-inflammatory genes as IL-1β in EAT from T2DM patients with CAD [29]. Also, rosiglitazone triggered lipid turnover and hypolipidemic actions by rapid browning of *WAT* in EAT depots of obese/T2DM rats by upregulation of PRDM-16, UCP-1 and mitochondrial biogenesis factors (i.e., PGC-1α, COX-4) [99] (Fig. 2). However, PPARγ activators have ben related with cardio-pathological secondary effects in T2DM patients [100].

Thus, only five anti-obesity drugs have been approved by the FDA for long-term treatments [101], but their role in VAT, and overall EAT, is rather unknown. An inhibitor of pancreatic lipase, orlistat, shrink 24% VAT volume in parallel to total cholesterol, LDL-C, TAG, and fasting blood glucose [102]. An agonist of serotonin receptor, lorcaserin, promoted weight loss in T2DM and non-diabetic mainly from the central region of the body [103]. Combination therapies may increase efficacy through synergistic actions, decreases adverse effects and increases tolerability. Thus, naltrexone + bupropion (opioid antagonist/amphetamine) demonstrated a reduction of body weight and VAT, and improved cardiovascular and metabolic parameters, such as blood pressure, lipids and glycaemia [104]. In overweight and obese/T2DM subjects, phentermine + topiramate (meta-amphetamine/monosaccharide) ameliorated body weight and obesity-associated cardio-metabolic conditions, such as blood pressure, total cholesterol and glycated haemoglobin levels [105]. In this sense, since statins have shown pleiotropic effects including the decrease of adipose tissue inflammation, they could also impact the *WAT* or *BAT* stores in EAT (Fig. 2). In fact, EAT thickness and inflammation were reduced in T2DM subjects and hyperlipidemic post-menopausal women after atorvastatin administration, independently of lipid lowering or CAD progression [106, 107].

Remarkably, agonists for glucagon-like-protein-1 receptors (GLP-1R) promoted insulin sensitivity, weight loss and adiponectin elevation in obese subjects. They also improved cardiovascular and metabolic parameters, such as blood pressure, lipids and glycaemia [108]. In particular, liraglutide shrink subcutaneous fat [109] and EAT (13%) in T2DM subjects after 12 weeks of treatment [110] (Table 2). In obese/T2DM individuals, liraglutide, but not metformin, reduced 29 and 36% EAT at 3 and 6 months, respectively, after administration [111]. Liraglutide also stimulated *WAT* browning and thermogenesis in mice independently of nutrient intake [112] (Fig. 2). Another GLP-1R agonist (exenatide), reduced EAT and subcutaneous and liver fat in T2DM patients, in a similar fashion than liraglutide [110]. Also, in obese rodents, exenatide induced a decrease of *WAT* in VAT and prompted plasma clearance of triacylglycerol and glucose, following *BAT* activation [112, 113]. In this line, sitagliptin, a DPP-4 inhibitor that avoid GLP-1 degradation, reduced also EAT (15%) in parallel to VAT and more intensively than BMI and waist circumference, in T2DM individuals [114]. Moreover, sitagliptin enhanced energy expenditure in obese mice by UCP-1 up-regulation in *BAT* repositories [115]. Thus, GLP-1R-associated effects may be also visceral fat specific, and stimulation of incretins could shift the energy balance from obesogenesis to thermogenesis. In this regard, the presence of functional extra-pancreatic GLP-1R has been reported in brain and adipose tissue [116, 117]. GLP-1R at the hypothalamus was crucial for *BAT* thermogenesis and *WAT* browning, as well as control of food intake [112, 115]. GLP-1R at the VAT and subcutaneous stores was found elevated in obese and T2DM patients with insulin resistance, where it participated in the overexpression of adiponectin [117, 118]. Finally, GLP-1 also triggered in vitro pre-adipocyte differentiation to stimulate adipocyte hyperplasia and insulin sensitivity [119]. Therefore, incretin may directly target VAT and EAT depots for fat regulation and insulin resistance in obese and T2DM patients.

## Conclusions

Adipose tissue may shift from being protective to being detrimental for obesity and cardiovascular homeostasis. *WAT* in VAT and EAT can hypertrophy and saturate in obese and T2DM subjects, becoming dysfunctional and releasing fatty acid and pro-inflammatory factors, in a positive feed-back loop. In this regard, some additional interventions to life-style change, such as bariatric surgery, *BAT* transplantation or anti-obesity drugs have exhibited promising outcomes on diminishing the *WAT*/*BAT* ratio. Nevertheless, further investigations are needed to discriminate whether this ratio can be specifically amended in EAT. Also, modifications of EAT transcriptome may open new avenues of treatment for cardiometabolic diseases. In these sense, PPARγ agonists and statins could impact on EAT depot by reduction of *WAT* thickness and pro-inflammation. More significant, incretin stimulation by GLP-1R agonists or DPP-4 inhibitors may reduce the obesogenic phenotype of *WAT* and encourage its trans-differentiation to *BAT*, either in VAT and EAT depots, leading to cardiovascular protection (Fig. 2). In addition, DDP4 inhibitors may also contribute to this action by their GLP-1-independent anti-inflammatory properties [120]. Thus, the incretin system may represent a *bona fide* candidate for improving fat deposition and distribution, and subsequent cardiovascular injuries, in obese and T2DM patients.
